# A novel multiplex polymerase chain reaction assay for the genotypic survey of the non-homologous end-joining factor 1 gene associated with Collie eye anomaly in Thailand

**DOI:** 10.14202/vetworld.2022.132-139

**Published:** 2022-01-25

**Authors:** Chommanad Lerdkrai, Nuch Phungphosop

**Affiliations:** Department of Physiology, Faculty of Veterinary Medicine, Kasetsart University, Bangkok, Thailand.

**Keywords:** Collie eye anomaly, dogs, multiplex polymerase chain reaction assay, non-homologous end-joining factor 1 genotype

## Abstract

**Background and Aim:**

Collie eye anomaly (CEA) is a hereditary and congenital ocular disorder, which affects several dog breeds, including Collies, Collie-related breeds, and other purebreds. An intronic deletion of 7799-bp in the non-homologous end-joining factor 1 (*NHEJ1*) gene has been identified as the genetic defect causing CEA. This study aimed to investigate the prevalence of CEA based on *NHEJ1* genotyping assay in Thailand.

**Materials and Methods:**

We clarified the prevalence of CEA in 224 dogs from five purebred dog breeds using a novel multiplex polymerase chain reaction (PCR)-based technique and confirmed the genotypic status with direct DNA sequencing.

**Results:**

The highest frequency of the mutated *NHEJ1* allele was 83.3% for Rough Collies, followed by 7.8% for Border Collies, 5.1% for Australian Shepherds, and 2.8% for Shetland Sheepdogs. The heterozygous mutated *NHEJ1* genotype detected for Rough Collies, Border Collies, Australian Shepherds, and Shetland Sheepdogs was 33.3%, 15.6%, 10.3%, and 3.3%, respectively. The homozygous mutated *NHEJ1* genotype was detected only in Rough Collies and Shetland Sheepdogs, accounting for 66.7% and 1.1%, respectively. Thai Ridgeback Dogs were not affected by this mutation.

**Conclusion:**

This study describes, for the 1^st^ time, the genotypic survey of the *NHEJ1* gene associated with CEA in dogs in Thailand. In addition, we successfully developed a new multiplex PCR assay with high accuracy, reproducibility, and cost-efficiency and validated its usefulness for determining *NHEJ1* genotypes.

## Introduction

Collie eye anomaly (CEA) is a canine hereditary ocular disease that involves the poor development of choroid and sclera, with varying manifestations. Overall, CEA is a congenital non-progressive disorder with asymmetrical bilateral lesions [1,2]. Choroidal hypoplasia in the temporal region to the optic disk is a typical clinical feature of CEA. Choroidal hypoplasia is characterized by a localized depletion of retinal and choroidal pigments combined with the absence of tapetal tissue, resulting in abnormal characteristics of choroidal blood vessels against the white atrophic background [2,3]. Besides choroidal hypoplasia, coloboma is also detected in CEA. This defect occurs due to improper closing of optic fissures during eye development and appears as a gray or pink pit of the optic disk surface or the adjacent area to the disk [2,4]. Additional clinical features, including intraocular hemorrhage, retinal detachment, and microphthalmos, may be observed in CEA. Most affected dogs retain normal visual function for their entire lives, whereas blindness can occur at a low prevalence in those with complete retinal detachment or intraocular hemorrhage [1-3].

CEA can be diagnosed accurately through ophthalmoscopic examination during 6-8 weeks of age. Beyond this period, the chorioretinal lesion is probably covered by retinal pigmentation, thus making the eye appear normal (so-called “go-normal phenomenon”) and resulting in misdiagnosis [1-3,5]. In 2007, Parker *et al*. [6] discovered the causative mutation underlying CEA by fine-mapping and mutation analysis. This study revealed that all affected breeds harbored a 7799-bp deletion mutation located in the intron 4 of the non-homologous end-joining factor 1 (*NHEJ1*) gene, which is considered an autosomal recessive trait. This finding provides an effective diagnostic tool for identifying CEA based on the *NHEJ1* genotyping. The first CEA case was reported in Smooth and Rough Collies, Border Collies, and Shetland Sheepdogs [1,2]. Several breeds, including Australian Shepherds, Lancashire Heelers, Australian Kelpies, Boykin Spaniels, Longhaired Whippets, Nova Scotia Duck Tolling Retrievers, Silken Windhounds, and Hokkaido dogs, have been diagnosed with CEA to date [6-12].

In Thailand, the population of herding dog breeds is currently growing compared to the previous decades. Nevertheless, CEA would probably remain unnoticed by dog owners, and *NHEJ1* genotypic testing is yet to become a part of the routine diagnosis of CEA in our country. To the best of our knowledge, no studies have been conducted in Thailand to determine the genotypic status of the *NHEJ1* gene, so the current situation of CEA and the distribution of *NHEJ1* genotypes in susceptible dog breeds and native Thai breed remain obscure.

This study determines CEA prevalence based on *NHEJ1* genotyping assay in a sample population of Rough Collies, Border Collies, Shetland Sheepdogs, Australian Shepherds, and Thai Ridgeback Dogs. Furthermore, we developed and validated the multiplex polymerase chain reaction (PCR)-based technique as an alternative diagnostic test for identifying *NHEJ1* genotypes.

## Materials and Methods

### Ethical approval

Ethical approval was obtained from the Kasetsart University Institutional Animal Care and Use Committee (Approval Number ACKU61-VET-095).

### Study period, location, animal, and sample collection

The study was conducted from January 2019 to February 2021, with target samples from the central part of Thailand. The peripheral blood-ethylenediaminetetraacetic acid (EDTA) samples were obtained from 224 clinically healthy dogs presented at Kasetsart University Veterinary Teaching Hospital. These dogs, ranging in age from 6 months to 10 years, and their *NHEJ1* genotypic status have not been previously assessed. The vision of the animals was tested by evaluating the menace response, the dazzle reflex, and the pupillary light reflex. We obtained all samples (with or without pedigree data) with informed consent from 74 private dog owners and seven breeding kennels, including one kennel for Border Collies, two kennels for Australian Shepherds, two kennels for Shetland Sheepdogs, and two kennels for Thai Ridgeback Dogs. Data from 21 Rough Collies, 90 Shetland Sheepdogs, 45 Border Collies, 39 Australian Shepherds, and 29 Thai Ridgeback Dogs were evaluated in this study.

### *NHEJ1* genotyping by multiplex PCR assay

**Figure-1:**
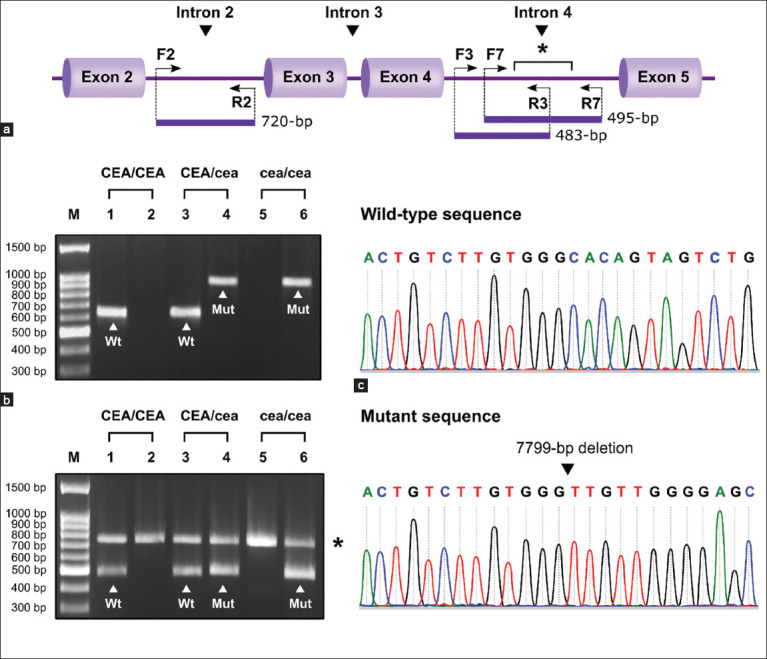
Genetic analysis of canine non-homologous end-joining factor 1 (*NHEJ1*) genotype. (a) Schematic diagram showing the binding sites of multiplex polymerase chain reaction (PCR) primers in the *NHEJ1* gene region and the expected size of the PCR products. Horizontal arrows represent the region and the direction of the wild-type allele-specific primers (F3 and R3), the mutant allele-specific primers (F7 and R7), and the internal control primers (F2 and R2). The asterisk (*) denotes a 7799-bp deletion mutation located in the intron 4. (b) Comparative evaluation of the conventional PCR assay and the multiplex PCR assay for *NHEJ1* genotyping. Upper panel: Agarose gel electrophoresis of the conventional PCR product. The white arrowheads indicate the 636-bp wild-type allele-specific and the 941-bp mutant allele-specific amplicons. Lower panel: Agarose gel electrophoresis of the multiplex PCR product. The white arrowheads indicate the 483-bp wild-type allele-specific and the 495-bp mutant allele-specific amplicons. The asterisk (*) corresponds to the 720-bp internal control amplicon of all samples. Lane M, 100-bp DNA molecular weight maker; lane 1-2: homozygous wild-type (CEA/CEA); lane 3-4: Heterozygous mutation (CEA/cea); Lane 5-6: Homozygous mutation (cea/cea) genotype. (c) Representative electropherograms of the *NHEJ1* gene sequence showing evidence of a 7799-bp deletion mutation (lower trace) compared to the wild-type sequence (upper trace). The black arrowhead designates a 7799-bp deleted site.

According to the standard protocol, total genomic DNA was extracted from fresh EDTA whole blood using the commercial QIAmp DNA Blood Mini Kit (Qiagen GmbH, Germany), and stored at –20°C until analysis. In the multiplex PCR assay, the target region of the canine *NHEJ1* gene was amplified by the specific primer sets located inside and outside the 7799-bp deletion mutation region (Figure-1a). These novel primer sets were designed based on GenBank accession number NC_006619.3, using the Primer-BLAST tool (https://www.ncbi.nlm.nih.gov/tools/primer -blast). As shown in Table-1, the wild-type primer set (F3 and R3) was designed to amplify a 483-bp fragment from the wild-type *NHEJ1* allele, whereas a 495-bp fragment was obtained from the mutated *NHEJ1* allele using the mutant primer set (F7 and R7). Furthermore, we constructed the internal control primer set (F2 and R2) and incorporated them into the wild-type and mutant primer sets. Therefore, this technique enabled the co-amplification of allele-specific products, along with the 720-bp fragment as an internal control. For the amplification of wild-type and mutated alleles in each sample, the multiplex PCR reaction required the use of two separate tubes. The first tube, containing a 25-μL volume of PCR mixture for detecting wild-type alleles, with a final concentration of 1× High Fidelity reaction buffer (10×, Invitrogen, USA), 0.2 mM each dNTP (Biotechrabbit, Germany), 2.0 mM MgSO_4_ (Invitrogen), 0.2 μM of F2 and R2 primers each (Biobasic Inc., Canada), 0.1 μM of F3 and R3 primers each (Biobasic Inc.), 1U Platinum^®^ *Taq* DNA Polymerase High Fidelity (Invitrogen), and 50-80 ng of template DNA. The second tube, containing the PCR mixture for detecting mutated alleles, consisted of similar components as in the first tube, except for the wild-type primer set, which was replaced by the mutant primer set. For detecting both wild-type and mutated alleles, we used a MiniAmp Plus Thermal Cycler (Thermo Fisher Scientific, USA) to perform the reaction procedure, which include a 5 min denaturation step at 95°C; followed by 35 cycles of 30 s at 95°C for denaturation, 2 min at 60°C for primer annealing, and 1 min at 72°C for extension, with a final elongation step for 10 min at 72°C. All amplified products were separated by size using electrophoresis on 1.5% agarose gel, stained with ethidium bromide, and subsequently visualized under ultraviolet illumination.

**Table-1:** Primer pair sequences and expected amplicon size for the multiplex polymerase chain reaction.

Primer set	Nucleotide sequence 5’ to 3’	Amplicon (bp)
Internal control primer set		
Forward primer (F2)	AATAAGGGCCGTGAGAGTGC	720
Reverse primer (R2)	GGCACCCGAAGCTATGTACC	
Wild-type allele primer set		
Forward primer (F3)	GTTTGCATGGTTGGAGAATCC	483
Reverse primer (R3)	AAGCTCAATCCTTAGAAGAGAAACC	
Mutant allele primer set		
Forward primer (F7)	CCTAGGAGTTTCATTTAGAATTCCC	495
Reverse primer (R7)	CACTGACAGCCTAATCTTGCC	

### *NHEJ1* genotyping by conventional PCR assay

In addition to the multiplex PCR assay, we performed the conventional PCR technique for determining the *NHEJ1* genotypes, as previously described [6]. In brief, we tested each sample in two separate reactions, which included two specific primer sets for wild-type and mutated alleles. To identify the wild-type allele, the first primer set, consisting of the forward primer (NHEJ1-F17) and the reverse primer (NHEJ1-R17), was used for amplification of a 636-bp fragment. For the mutated allele, the second primer set, consisting of the forward primer (NHEJ1-F20) and the reverse primer (NHEJ1-R23), was employed for amplification of a 941-bp fragment (Table-2) [6]. All PCR reactions were prepared in a final reaction volume of 25-mL consisting of 1× High Fidelity reaction buffer (10×, Invitrogen), 0.2 mM each dNTP (Biotechrabbit), 2.0 mM MgSO_4_ (Invitrogen), 0.1 mM each primer (Biobasic Inc.), 1U Platinum^®^ *Taq* DNA Polymerase High Fidelity (Invitrogen), and 50-80 ng of template DNA. The amplification reactions for both alleles were performed with a 5 min denaturation step at 95°C, 35 reaction cycles of 30 s at 95°C for denaturation, 1 min at 59°C for primer annealing, and 1 min at 72°C for extension, with a final elongation step for 10 min at 72°C in a MiniAmp Plus Thermal Cycler (Thermo Fisher Scientific). Electrophoresis and visualization of the amplified PCR products were performed as described above.

**Table-2:** Primer pair sequences and expected amplicon size for the conventional polymerase chain reaction [6].

Primer set	Nucleotide sequence 5’ to 3’	Amplicon (bp)
Wild-type allele primer set		
Forward primer (NHEJ1-F17)	TCTCACAGGCAGAAAGCTCA	636
Reverse primer (NHEJ1-R17)	CCATTCATTCCTTTGCCAGT	
Mutant allele primer set		
Forward primer (NHEJ1-F20)	TGGGCTGGTGAACATTTGTA	941
Reverse primer (NHEJ1-R23)	CCTTTTTGTTTGCCCTCAGA	

### Sequencing

A total of 224 samples were submitted for direct DNA sequencing to confirm *NHEJ1* genotypes. The wild-type primer set (F3 and R3) and the mutant primer set (F7 and R7) were used to amplify a fragment harboring the wild-type and the mutated allele, respectively. The PCR amplification reactions were conducted as described above. The amplified PCR products were purified with a NucleoSpin^®^ Gel and PCR Clean-up purification kit (Macherey-Nagel, Germany) according to the manufacturer’s instruction and then sequenced by Macrogen Inc. (Korea) using the forward primers (F3 and F7). Sequence data were analyzed using the SnapGene^®^ Viewer program (SnapGene^®^ Software, GSL Biotech LLC, USA). The sequence similarity was compared with the National Center for Biotechnology Information database (NCBI; https://blast.ncbi.nlm.nih.gov/blast.cgi).

## Results

The novel multiplex PCR assay developed in this study constituted three different primer sets for the identification of the canine *NHEJ1* genotypes (Figure-1a). Agarose gel electrophoresis of the amplified PCR products derived from this assay revealed the three different fragment patterns, allowing discrimination of the *NHEJ1* genotypes into healthy or homozygous wild-type (CEA/CEA), carrier or heterozygous mutation (CEA/cea), and affected or homozygous mutation (cea/cea). In healthy dogs carrying two copies of the wild-type allele, a single 483-bp fragment was amplified from the wild-type primer set, while a single 495-bp fragment obtained from the mutant primer set was detected in affected dogs harboring two copies of the mutated allele. Furthermore, these two allele-specific fragments were simultaneously observed in carrier dogs exhibiting the heterozygous genotype for this mutation. Besides the allele-specific fragments, the expected fragment of 720-bp, corresponding to the internal control, was amplified uniformly for any genotype sample with similar intensity and clearly separated from the allele-specific fragments in the gel image (Figure-1b). The results obtained from the newly developed technique were 100% concordant with those from the original conventional PCR assay [6] and direct DNA sequencing.

Table-3 shows the results of *NHEJ1* genotypes and allele frequencies of 224 dogs. Of the five purebred dog breeds examined in this survey, the *NHEJ1* mutation was detected only in four potentially affected dog breeds, namely, Rough Collies, Border Collies, Australian Shepherds, and Shetland Sheepdogs. Thai Ridgeback Dogs were not affected by this mutation. Among the affected breeds, the remarkably high frequency of *NHEJ1* mutation at 83.3% was observed in Rough Collies. In comparison, the remaining Collie-related breed presented significantly low mutation frequencies of 7.8% for Border Collies, 5.1% for Australian Shepherds, and 2.8% for Shetland Sheepdogs. In Rough Collies, all tested dogs harbored at least one copy of the mutated *NHEJ1* allele, in which the prevalence of the cea/cea genotype was approximately two-fold higher than those of the CEA/cea genotype, accounting for 66.7% and 33.3%, respectively. In Shetland Sheepdogs, approximately 95.6% of the tested dogs exhibited intact *NHEJ1* genotype, whereas the remaining individuals presented either the CEA/cea or the cea/cea genotype, with a prevalence of 3.3% and 1.1%, respectively. In Border Collies and Australian Shepherds, most of these dogs presented the CEA/CEA genotype, accounting for 84.4% and 89.7%, respectively. The mutated cases in these two breeds predominantly exhibited the CEA/cea genotype, with the prevalence of 15.6% for Border Collies and 10.3% for Australian Shepherds.

**Table-3:** Table of *NHEJ1* genotypes and allele frequencies in 224 dogs of five breeds in Thailand.

Breed	N	Genotype frequencies			Allele frequencies (%)	
	
		CEA/CEA n (%)	CEA/cea n (%)	cea/cea n (%)	CEA	cea
Rough Collie	21	0 (0)	7 (33.3)	14 (66.7)	16.7	83.3
Border Collie	45	38 (84.4)	7 (15.6)	0 (0)	92.2	7.8
Australian Shepherd	39	35 (89.7)	4 (10.3)	0 (0)	94.9	5.1
Shetland Sheepdog	90	86 (95.6)	3 (3.3)	1 (1.1)	97.2	2.8
Thai Ridgeback Dog	29	29 (100)	0 (0)	0 (0)	100	0

CEA/CEA=Homozygous wild-type genotype, CEA/cea=Heterozygous mutation genotype, cea/cea=Homozygous mutation genotype, N=Number of examined dogs of each breed, n=Number of dogs of each genotypic pattern

**Figure-2:**
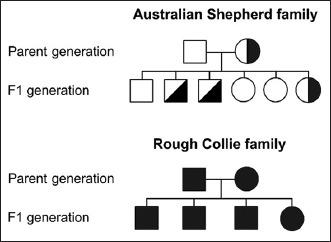
The partial pedigree charts of *NHEJ1* mutation in the Australian Shepherd family (upper panel) and the Rough Collie family (lower panel). Circles and squares represent females and males, respectively. Healthy individuals (CEA/CEA genotype) are depicted with open symbols. Carrier individuals (CEA/cea genotype) are depicted with half-filled symbols. Affected individuals (cea/cea genotype) are depicted with filled-in symbols.

The partial pedigree charts of the two families are illustrated in Figure-2. In the Australian Shepherd family, the pedigree was comprised of two generations with eight individuals. Mating between the CEA/CEA genotype sire and the CEA/cea genotype dam produced F1 progeny with 50% CEA/CEA genotype (one female and two males) and 50% CEA/cea genotype (two females and one male). For the Rough Collie family, the pedigree was comprised of two generations with a total of six individuals. Mating between the cea/cea genotype sire and dam produced all affected F1 progeny (three males and one female). This finding indicates that the transmission of *NHEJ1* mutation across generations is not likely to be a gender predilection. The transmission pattern shows the classic feature of Mendelian inheritance of an autosomal recessive trait.

## Discussion

The diagnosis of CEA by conventional PCR-based canine *NHEJ1* genotyping was first introduced by Parker *et al*. [6]. To date, several molecular genotyping assays for detecting *NHEJ1* genotypes have been developed, including a rapid direct PCR amplification method without DNA extraction [13] and real-time PCR based on SYBR Green combined with Flinders Technology Associates cards [14]. In this study, we successfully established a novel multiplex PCR assay for identifying *NHEJ1* genotypes in five purebred dog breeds in Thailand. According to the conventional PCR protocol [6], the wild-type and mutant allele-specific amplicon were directly differentiated by size on gel; however, the false-negative results may occur if this technique was improperly done. To mitigate this, the multiplex PCR assay, consisting of the allele-specific primer sets and the internal control primer set, was developed in this study. Based on this assay, we designed the specific wild-type and mutant primer set to encompass the 7799-bp deletion mutation of intron 4. In addition, the internal control primer set, corresponding to the amplification of a conserved region at intron 2, was included for monitoring successful PCR amplification and minimizing the likelihood of false-negative results. The amplified PCR products were generated, yielding the specific amplicon for wild-type and mutated alleles along with the internal control fragment. As shown in Table-3, all genotypes of the *NHEJ1* gene analyzed in 224 dogs were determined using this multiplex PCR assay. The obtained results were consistent with the gold-standard DNA sequencing and the original conventional PCR assay [6]. Thus, the method proposed here is a simple and highly reliable diagnostic tool for precisely detecting *NHEJ1* genotypes.

Moreover, this technique requires basic genetic analysis instruments without expensive equipment and high-cost reagents compared to other molecular approaches. Although this novel protocol enables us to overcome the limitation of the conventional PCR protocol, the major drawback of our technique is that the wild-type and mutant allele-specific products are very close in size compared to those obtained from the previous protocol. Thus, it is critical to include an appropriate positive and negative control sample in each PCR run to ensure the accuracy of results.

In the last decade, the ophthalmoscopic examination has been extensively used as a primary diagnostic method for CEA in various studies. The distribution of CEA obtained from those surveys varied across affected breeds. Regarding the large-scale studies in Rough and Smooth Collies, the overall prevalence of affected dogs was approximately 30.95%–40.6% in Finland [15], Switzerland [16], Norway [17], and the Netherlands [18]. In addition, a high prevalence of about 64% and 75.5% was observed in the UK [5] and Denmark [19], respectively. Furthermore, the results obtained for Collie’s populations in the USA were significantly higher than those reported for the same breed in European countries, where the prevalence ranged from 79.9% to 90% [1]. In Shetland Sheepdogs, a wide range of prevalence was identified across different studies in the European zone. As stated in large population studies in Switzerland [16] and Germany [20], the prevalence of affected dogs was estimated to be 15.1% and 19.7%, respectively. In addition, relatively high prevalence, ranging from 48.3%–50%, was reported in the Netherlands [1] and Denmark [19]; values as high as 72% were observed in the UK [5]. Considering CEA occurrence in Australian Shepherds, the prevalence of affected individuals was only 0.17% in Switzerland [16], and 4.03% in Australia [21]. Similarly, a low prevalence of CEA has been demonstrated in Border Collies. A prevalence of 0.7% was reported in Switzerland [16], and a slightly high prevalence of 6% was detected in the UK [22]. Based on these investigations, the highest prevalence of CEA was observed in Collies, and the second-most affected breed was the Shetland Sheepdogs, whereas the Border Collies and the Australian Shepherds are less affected. Apart from the potentially affected dog breeds, the prevalence of CEA was described in the Lancashire Heeler in the UK [23] and the Hokkaido Dogs in Japan [8], with prevalence’s of 13.7% and 29.4%, respectively.

Although *NHEJ1* genotyping assay was available for CEA diagnosis, the geographic distribution of the *NHEJ1* genotypic status across different countries remains undiscovered. In the Rough Collies, a remarkably high mutation frequency of 83.3% was observed in this study; this value is slightly higher compared to that reported in the Czech Republic, where the mutation frequency was 79.7% [13]. In Denmark, however, a considerably higher mutation frequency of 98.89% was reported [19]. Interestingly, all tested individuals in the previous studies harbored at least one copy of the mutated *NHEJ1* allele, and a similar trend was observed in our survey. Concerning earlier reports and this study, the occurrence of the cea/cea genotype predominated over the CEA/cea genotype. The mutation frequency in the *NHEJ1* gene was significantly high for the Rough Collies compared to other Collie-related breeds. Almost all Shetland Sheepdogs tested in this survey had an intact *NHEJ1* gene, and both *NHEJ1* deletion patterns were detected in some individuals, with a frequency of 2.8%. This finding was extremely low compared to that described for the Czech Republic [13] and Denmark [19], where the mutation frequencies were 42.9% and 58.9%, respectively. Among the mutated cases examined in these investigations, the CEA/cea genotype was more frequent, except in Denmark, where the cea/cea genotype predominated [19]. In the Border Collies, our survey revealed that most tested dogs exhibited the CEA/CEA genotype, whereas a small proportion of individuals exhibited the mutation (7.8%). This finding is closely related to the results of similar studies from Norway [24] and Belgium [25], where the mutation frequencies were 6.3% and 8.9%, respectively. However, higher mutation frequencies of 14.3% in Japan [26] and 19.4% in the Czech Republic [13] were observed. According to most of these earlier studies, the prevalence of the CEA/cea state was more frequent in the mutated cases, and this trend is similar to the pattern detected in this survey. We discovered that the common *NHEJ1* genotypic pattern in Australian Shepherds, as in Border Collies, was the intact state, with only 5.1% having the mutation. Similar findings were reported from Belgium [25] and the Czech Republic [13], where the mutation frequencies were 3.1% and 4.5%, respectively. However, a slightly higher mutation frequency of 11% has been observed in a large-scale study in Australian Shepherds originating from Europe and the USA [27]. Among Australian Shepherds with the *NHEJ1* mutation, the CEA/cea genotype predominated in all mutated individuals, whereas the cea/cea genotype was unidentified across previous studies and in this investigation. Regarding the Thai Ridgeback Dogs, this Thai native breed is a hunting dog, as described by the Fédération Cynologique Internationale, and is not a descendant of the Collie lineage. As expected, none of the cases of Thai Ridgeback Dogs harbored this mutation. Concerning the *NHEJ1* genotypic survey from several studies, a high occurrence of *NHEJ1* mutation is observed in the Rough Collies. The second-most affected breed is the Shetland Sheepdog, whereas the Border Collies and Australian Shepherds exhibit relatively low *NHEJ1* mutation rates. Although these observations were conducted with a limited number of dogs, the overall results provide the striking finding that the trend of *NHEJ1* mutation in prone dog breeds is closely related to that determined in the clinical phenotypic survey.

The phenotypic transmission trait of CEA was first investigated in the USA in 1968 byYakely *et al*. [28]. Based on a test mating study in Collies, the authors concluded that the transmission of CEA symptoms among generations followed an autosomal recessive trait and was not a sex-linked pattern. This finding was supported by a later study in the UK using the analysis of pedigree data and test mating in a large population of Rough Collies, Smooth Collies, and Shetland Sheepdogs [5]. However, contradictory results were observed in Sweden. Regarding the comparison of CEA symptoms in the offspring and their related parents in Collies, the authors observed that the phenotypic transmission pattern of CEA was not in concordance with a simple autosomal recessive inheritance [29]. In addition, the non-Mendelian phenomenon of CEA was reported in the USA. According to a segregation study in Rough Collies, Smooth Collies, Border Collies, Australian Shepherds, and Shetland Sheepdogs, the intergenerational transmission of CEA phenotype in affected individuals revealed an incomplete penetrance pattern [30].

Furthermore, the partial penetrance pattern was observed for some heterozygous individuals [30]. As the genetic mutation causing CEA has been established, the compliance between the clinical phenotype and *NHEJ1* genotypic status was investigated in some countries. As stated in a study on Rough Collies and Shetland Sheepdogs in Denmark, there was a correlation between clinical symptoms and *NHEJ1* genotypes in the Shetland Sheepdog population; however, poor compliance was discovered in the Rough Collie population [19]. Recently, an investigation in Border Collies in Norway demonstrated that the clinical diagnosis and *NHEJ1* genotype testing perfectly matched in all examined puppies [24]. In this survey, a clinical phenotypic assessment was excluded, and thus, the genotype-phenotype association of CEA was undetermined. However, we evaluated the visual function of individuals using a basic neuro-ophthalmological examination. As per the results obtained from this evaluation, all examined dogs with or without mutated *NHEJ1* allele exhibited a positive response to all visual tests, making it reasonable to assume that they possess normal vision. This is unsurprising since visual impairment and blindness rarely occur in CEA.

As previously reported by Lowe *et al*.[30], CEA is a complex disease with various symptoms, and the underlying cause is probably associated with mutations in multiple genes; thus, CEA diagnosis is not straightforward. So far, combining the clinical phenotype evaluation and the *NHEJ1* genotypic assay could facilitate the effectiveness of the diagnostic procedure. A timely CEA diagnosis is critical for ophthalmoscopic examination. Clinical assessment should be performed by an expert veterinarian, and the most reliable test should be done between the ages of 6 and 8 weeks to avoid the go-normal phenomenon. Screening of the *NHEJ1* genotype, in addition to the clinical phenotype of individuals, should be evaluated to identify the disease allele. The potential benefit of the genotypic assay is the discrimination of the carrier from wild-type and affected individuals. Information regarding the clinical phenotype and the *NHEJ1* genotypic status could be an efficient tool for breeders to develop adequate breeding strategies.

## Conclusion

In this study, we successfully developed a novel multiplex PCR-based technique for determining the *NHEJ1* status and validating its usefulness for the genotypic survey of the *NHEJ1* gene associated with CEA in the most prone herding and native breeds in Thailand. Concerning this survey, we emphasize that identifying the *NHEJ1* genotype should be interpreted with caution since the clinical features nor the symptom severity of CEA could be determined in this study. To better understand the distribution of this disease in Thailand, more research should be conducted to investigate the relationship between the clinical phenotype of CEA and the genotypic status of the *NHEJ1* gene.

## Authors’ Contributions

CL: Examined animal’s clinical status, collected blood samples, designed the study, conducted the experiments, analyzed data, drafted, reviewed, and edited the manuscript. NP: Provided laboratory technical support. All authors read and approved the final manuscript.
